# Histone deacetylase inhibitors containing a benzamide functional group and a pyridyl cap are preferentially effective human immunodeficiency virus-1 latency-reversing agents in primary resting CD4^+^ T cells

**DOI:** 10.1099/jgv.0.000716

**Published:** 2017-04-27

**Authors:** Yoshifumi Kobayashi, Céline Gélinas, Joseph P. Dougherty

**Affiliations:** ^1^​ Department of Molecular Pharmacology, Rutgers Robert Wood Johnson Medical School, Piscataway, NJ 08854, USA; ^2^​ Center for Advanced Biotechnology and Medicine and the Department of Biochemistry and Molecular Biology, Rutgers Robert Wood Johnson Medical School, Piscataway, NJ 08854, USA

**Keywords:** Epigenetics, HDAC inhibitor, HIV, primary resting T cell, latency-reversing agents

## Abstract

Antiretroviral therapy (ART) can control human immunodeficiency virus-1 (HIV-1) replication in infected individuals. Unfortunately, patients remain persistently infected owing to the establishment of latent infection requiring that ART be maintained indefinitely. One strategy being pursued involves the development of latency-reversing agents (LRAs) to eliminate the latent arm of the infection. One class of molecules that has been tested for LRA activity is the epigenetic modulating compounds histone deacetylases inhibitors (HDACis). Previously, initial screening of these molecules typically commenced using established cell models of viral latency, and although certain drugs such as the HDACi suberoylanilide hydroxamic acid demonstrated strong activity in these models, it did not translate to comparable activity with patient samples. Here we developed a primary cell model of viral latency using primary resting CD4^+^ T cells infected with Vpx-complemented HIV-1 and found that the activation profile using previously described LRAs mimicked that obtained with patient samples. This primary cell model was used to evaluate 94 epigenetic compounds. Not surprisingly, HDACis were found to be the strongest activators. However, within the HDACi class, the most active LRAs with the least pronounced toxicity contained a benzamide functional moiety with a pyridyl cap group, as exemplified by the HDACi chidamide. The results indicate that HDACis with a benzamide moiety and pyridyl cap group should be considered for further drug development in the pursuit of a successful viral clearance strategy.

## Abbreviations

ART, antiretroviral therapy; HAT, histone acetyltransferase; HDAC, histone deacetylase; HDACi, histone deacetylase inhibitor; HIV, human immunodeficiency virus; HMT, histone methyltransferase; HMTi, histone methyltransferase inhibitor; LRA, latency-reversing agent; MCF, mean channel fluorescence; PEI, polyethyleneimine; PMA, phorbol 12-myristate 13-acetate; SAHA, suberoylanilide hydroxamic acid; SIV, simian immunodeficiency virus.

## Introduction

It is estimated that there are 36.7 million people infected with human immunodeficiency virus (HIV) worldwide [1]. Although antiretroviral therapy (ART) has been very successful in managing infection, it does not result in a cure. Extensive research indicates that the major obstacle to clearing the virus is the early establishment of a latently infected population of cells that serve as a life-long source for viral rebound [2]. Latent infection is a reversible non-productive infection, which does not preclude some virus expression in the absence of virion production [3]. Moreover, it has been demonstrated that resting memory CD4^+^ T cells represent an important component of the latent reservoir [4].

Development of a strategy to eliminate the latent component of the infection is an important challenge. One idea is to identify latency-reversing agents (LRAs) and use them to eliminate the latent arm of the infection. The strategy is dependent upon killing of virus-producing cells by a combination of viral protein-induced toxicity and immune surveillance as well as concomitant ART treatment to prevent infection of bystander cells. As an adjunctive therapy, it may also be useful to boost immune killing by priming against viral antigens [5].

Numerous epigenetic modifier proteins have been shown to affect HIV-1 latency [6]. For example, histone deacetylases (HDACs) 1, 2 and 3 are involved in establishing and maintaining HIV-1 latency [7]. The histone acetyltransferases (HATs) p300/CBP and P/CAF are recruited to the HIV-1 promoter by NFκB [8]. Histone methyltransferases (HMTs), including SUV39H1 [9], G9a [10] and EZH2 [11], have also been reported to affect HIV-1 latency. DNA (cytosine-5-)-methyltransferase 1 (DNMT1) methylates CpG islands at the HIV-1 LTR [12]. The PBAF SWI/SNF chromatin remodelling complex activates HIV-1 transcription while BAF, another SWI/SNF complex, inhibits HIV-1 transcription [13]. Many of these modifiers can change the chromatin structure of the nucleosomes nuc-0 and nuc-1 that form on the 5′-LTR, altering accessibility of transcription factors to this region affecting transcription and viral latency [14].

Compounds targeting epigenetic modifiers have been investigated for LRA activity. In particular, a few HDAC inhibitors (HDACis) have been advanced for clinical trials. For example, clinical trials have been performed with the HDACis suberoylanilide hydroxamic acid (SAHA), valproic acid and panobinostat, and although treatment with these compounds led to an increase in viral RNA expression, they seemed to lack sufficient potency to promote significant viral clearance [15–17]. This suggests that it is important to continue to search for more potent epigenetic modulating compounds that could also be used in combination therapy.

Here we evaluate 94 epigenetic modulating compounds. The screen was performed with a primary cell model of HIV-1 latency that closely mimics the activation profile obtained using patient samples treated with previously identified LRAs. HDACis yielded the greatest activity. In particular, HDACis with a benzamide functional moiety and pyridyl-cap group displayed greater efficacy, as exemplified by chidamide. The results suggest that HDACis such as chidamide should be considered for further drug development in the quest for a viable viral clearance strategy.

## Results

### Primary resting CD4^+^ T cell model of latency complemented with Vpx

**Fig. 1.**
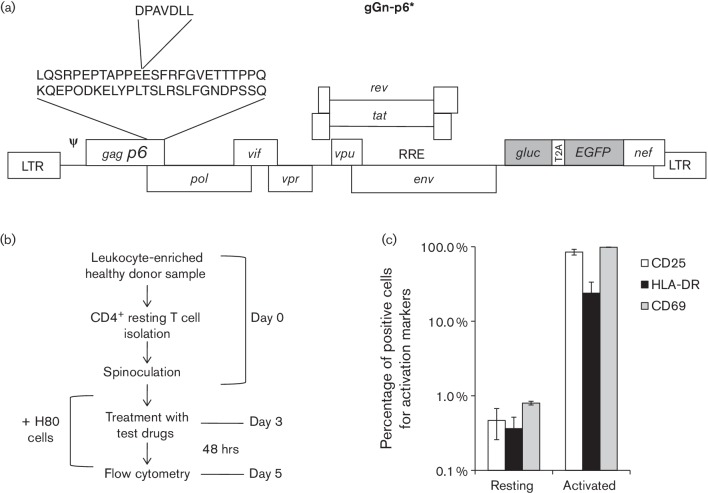
Protocol for establishing latent virus infection in primary resting CD4**^+^** T cells. (a) Construct gGn-p6* is a replication competent vector with the Vpx binding motif (DPAVDLL) inserted within p6 as indicated. *gluc*, *Gaussia* luciferase gene; T2A, T2A peptide promoting ‘ribosomal skipping’; *EGFP*, enhanced green fluorescent protein gene. (b) Primary resting CD4^+^ T cells were isolated from leukocyte-enriched healthy donor samples using a Ficoll gradient and negative selection. The isolated T cells were infected by spinoculation with gGn-p6* virus containing Vpx protein. Three days following infection, cultures were treated with test compounds followed 48 h later by flow cytometric analyses. Where indicated, resting cells were co-cultured with the H80 glioma cell line at day 1 after infection. (c) Percentage of activation-marker positive cells in isolated resting CD4^+^ T cells. Cells from three independent healthy donors or those cells activated with anti-CD3/CD28 beads (Life Technologies) were stained with FITC-conjugated antibodies for each marker and analysed via flow cytometry. Each column shows the mean of three experiments. Error bars show standard deviations.

**Fig. 2.**
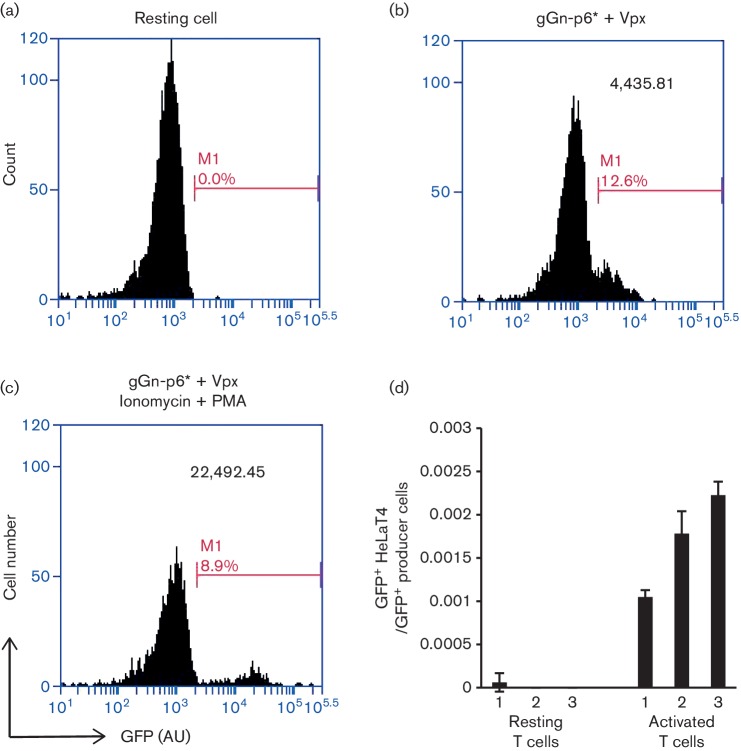
Evaluation of the primary cell model. (a) Flow cytometric analysis of uninfected resting cells. (b) Flow cytometric analysis of cells infected with gGn-p6* virus. (c) Flow cytometric analysis of infected cells treated with ionomycin and PMA. (d) Low-level virus production from resting cells infected with gGn-p6*. HeLaT4 cells were inoculated with supernatants from gGn-p6* infected resting T cells or activated T cells. The number of infected GFP^+^ HeLaT4 cells was measured via flow cytometry and normalized to the number of GFP^+^ virus producer cells (resting cells or activated cells). Each mean was calculated from triplicate samples from three independent blood donors 1–3, as indicated on the horizontal axis. Error bars show standard deviations. AU, arbitrary unit.

To evaluate epigenetic modulating compounds for latency reversing activity, a primary resting CD4^+^ T cell model of latency was developed. Advantage was taken of the finding that simian immunodeficiency virus (SIV)/HIV-2 encoded Vpx has the ability to overcome the restriction to infection posed by SAMHD1, which hydrolyses dNTPs needed for HIV-1 reverse transcription [18]. It was reasoned that complementing HIV-1 with Vpx might promote more efficient infection of primary resting CD4^+^ T cells, and therefore more effective establishment of latent infection. To this end, the HIV-1-derived vector gGn-p6* was constructed (Fig. 1a). The Vpx binding motif, DPAVDLL [19], was inserted into the p6 gene of gGn-p6* in order to direct the incorporation of Vpx into assembling virions produced from cells co-transfected with pgGn-p6* and pcDNA-VPXsiv, a Vpx-expressing plasmid (Fig. 1a) [19–21]. Primary resting CD4^+^ T cells from healthy donors were then infected with gGn-p6* virus complemented with Vpx (Fig. 1b). It is noteworthy that the purified resting CD4^+^ T cells contained fewer than 1 % activated cells as determined by assessing the percentage of cells expressing the CD25, CD69 or HLA-DR activation markers (Fig. 1c). The infection efficiency ranged between at least 2 and 15 % as indicated by GFP expression (Fig. 2a, b). The efficiency of infection was similar to the value previously reported by Baldauf *et al.* [22]. Moreover, activation of latent virus by treatment with ionomycin and phorbol 12-myristate 13-acetate (PMA) resulted in an approximately fivefold increase in GFP mean channel fluorescence (MCF) (Fig. 2c). For subsequent experiments to test LRAs, we maintained the infected primary T cells on a feeder layer of H80 cells, a U251MG glioma cell line. Using the H80 feeder cells promoted survival of 90 % of the primary resting T cells up to 10 days, whereas only 40 % survived without the H80 feeder cells [23, 24] (data not shown). Although at least some of the infected primary resting T cells expressed sufficient GFP for detection, they did not produce virus, indicating that they established a latent infection (Fig. 2d).

### Latent virus activation profile by LRAs is similar for the primary cell model and patient samples

**Fig. 3.**
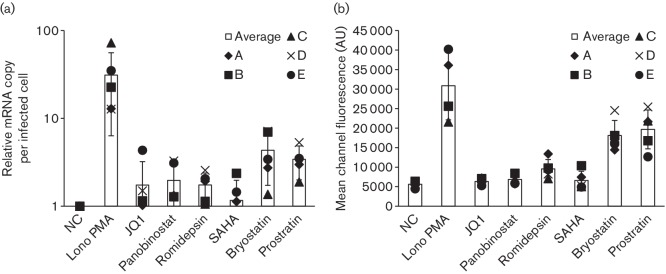
Activation pattern of latent virus in the primary cell model using previously described LRAs. Resting T cells infected with gGn-p6* were treated with 400 nM ionomycin plus 20 ng PMA ml^−1^, 1 µM JQ1, 30 nM panobinostat, 40 nM romidepsin, 335 nM SAHA, 10 nM bryostatin or 300 nM prostratin. Relative mRNA copy number (a) and GFP mean channel fluorescence (b) from the infected cells were measured 24 or 48 h later, respectively. Relative mRNA copy number per infected cell was determined by dividing the value from quantitative PCR by the GFP-positive cell number ascertained via FACS analysis. Each value is obtained from four or five healthy donor samples indicated with symbols. NC, negative control. Error bars show standard deviations. AU, arbitrary unit.

To test the relevance of the primary cell model described above to latent infection of patient cells, latently infected primary resting CD4^+^ T cells were treated with a panel of previously described LRAs to determine if the pattern of activation obtained with the model system was similar to that obtained with patient samples. Latently infected cells were treated with the LRAs JQ1 [25], panobinostat (Novartis, 2007), romidepsin (Astellas Pharma, 1994), SAHA [26], bryostatin [27] and prostratin [28], with ionomycin (Meyers, E, US patent, 1975) plus PMA [29] serving as a positive control. JQ1 is a BET-family protein inhibitor. Bryostatin and prostratin are protein kinase C agonists while SAHA, panobinostat and romidepsin are HDACis. As indicators of LRA activity, gGn-p6* RNA was quantified and GFP MCF was measured 24 and 48 h, respectively, after treatment with the indicated LRAs (Fig. 3a, b). Ionomycin plus PMA drastically activated latent virus with a 10–70-fold increase in RNA levels and with a 4–8-fold increase in GFP levels. Bryostatin and prostratin also notably activated HIV with a 1.4–7.0-fold increase in RNA levels and a 2–5-fold increase in GFP expression. Activation with JQ1 and the three HDACis was relatively minor. The activation pattern obtained with this primary cell system is very similar to that obtained with patient samples as previously reported [30]. For example, it was reported that SAHA is not very effective at activating latent virus from patient samples while bryostatin-1 did reactivate virus [30], which was also the case with the our latency model (Fig. 3a, b). Moreover, the same reactivation pattern extended to the other LRAs with our model system, suggesting that this model should be useful for further drug development.

### Evaluation of a panel of epigenetic modulating compounds employing a primary cell model of latency

**Fig. 4.**
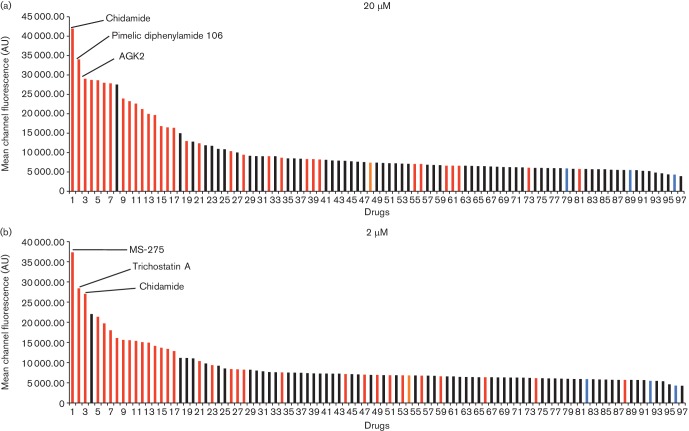
Distribution of EGFP MCF from gGn-p6*-infected resting T cells treated with drugs in the Epigenetics Screening Library. EGFP MCF from infected resting cells treated with drugs is depicted in descending order. The data were obtained using cells isolated from three donors. Numbers on the *x*-axis indicate ranking position of the compounds. Red, blue and orange columns indicate HDACi, negative control and SAHA, respectively. The top three activators are noted in each graph. AU, arbitrary unit.

To determine the efficacy of epigenetic modulating compounds as LRAs in primary resting CD4^+^ T cells, 94 chemical compounds from the Epigenetics Screening Library (Cayman Chemical) were tested for LRA activity using the primary cell latency model described above. Cultures were treated with each compound at two concentrations, 2 and 20 µM, for 48 h. MCF was then measured for each sample. Interestingly, 16 out of 34 HDACis were among the 17 best activators at both concentrations, indicating that HDACis represent the most effective cluster among epigenetic compounds for LRA activity (Fig. 4a, b). Again, SAHA, the HDACi that was quite effective in established cell models of latency, was not effective with the primary cell model as was also previously found with patient samples (Fig. 4a, b) [30, 31].

The three most potent activators at 2 µM were MS-275, chidamide and trichostatin A, while at 20 µM they were chidamide, pimelic diphenylamide 106 and AGK2. Interestingly, there is a concentration of HDACis containing benzamide functional moieties among the most potent hits (Table 1). In the library of 94, there are only four HDACis containing benzamide groups, including chidamide, pimelic diphenylamide 106, MS-275 and CAY10433. Three of four benzamide-containing HDACis scored among the top three activators at 2 and/or 20 µM, indicating that it would be prudent to focus upon benzamide-containing HDACis for further drug development. This is further supported by the finding that chidamide was among the most potent LRAs at both 2 and 20 µM (Fig. 4).

**Table 1.** HDACis which showed highest potency in resting T cells

Activator	Structure	Mean channel fluorescence	
		20 µM	2 µM
Chidamide	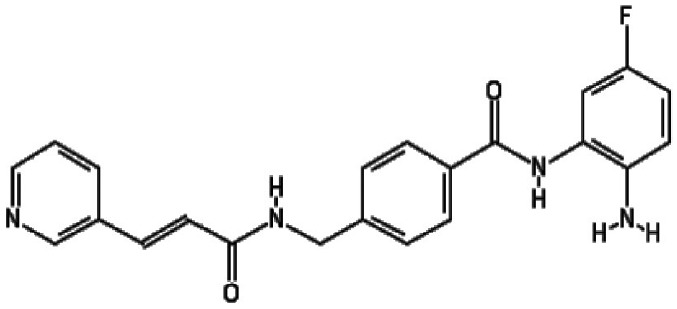	41 957.68	27 034.24
MS-275	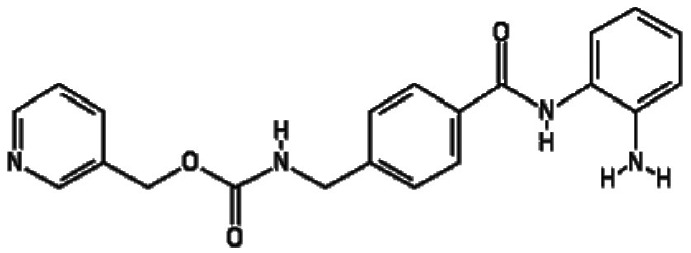	34 004.80	12 861.62
Pimelic diphenylamide 106	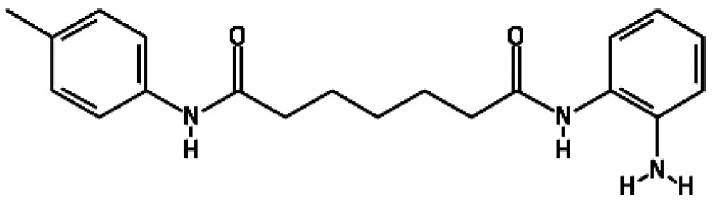	23 247.27	37 335.96

**Fig. 5.**
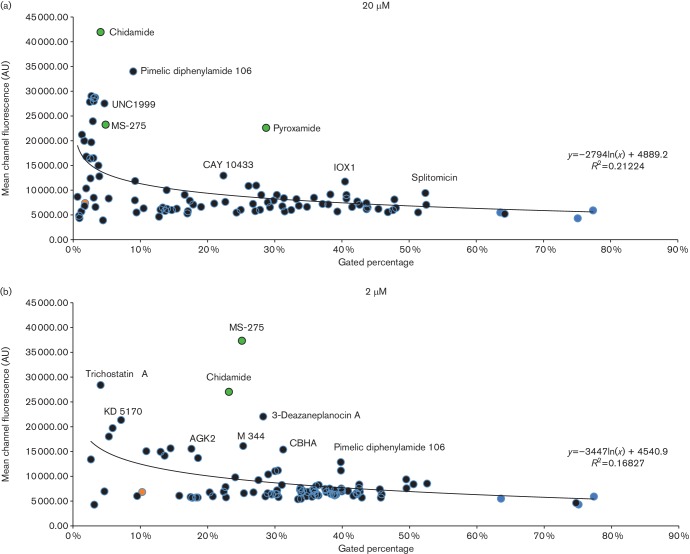
Scatter plot of MCF and gated cell ratio from infected resting T cells treated with drugs in the Epigenetics Screening Library. Graphs from cells treated with drugs at 20 µM (a) and 2 µM (b). The data were obtained using cells isolated from three donors. MCF data and the gated ratio of resting cells are indicated on the *y*-axis and *x*-axis, respectively. Modulator drugs that plotted distantly from each graph’s trend line are noted. Green, orange and light blue circles indicate HDACis with a pyridine cap group, SAHA and negative controls, respectively. AU, arbitrary unit.

To assist in further prioritizing ‘hits’, the cellular toxicity of HIV-activating drugs was examined. Flow cytometric analysis can be used to distinguish living cells from dead cells or debris by gating samples utilizing forward scatter and side scatter [32]. Therefore, drug toxicity was initially monitored by measuring the ‘gated’ cell ratio obtained during flow cytometric analysis. Next, the percentage of gated living cells was plotted against the MCF obtained for each molecule to assist in focusing upon the more promising ‘hits’ (Fig. 5a, b). Additionally, the (MCF) × (gated percentage) was calculated to serve as an indicator of the most promising ‘hits’ because it is proportional to both cell survival and drug efficacy. Fig. 5 and the (MCF) × (gated percentage) indicated that the three top candidates are chidamide, MS-275 and pyroxamide, all of which interestingly contain pyridine caps (Table 2). Lastly, the CC_50_ values for chidamide, MS-275 and pyroxamide were determined by counting dead cells stained with propidium iodide using FACS analysis and were found to be comparable to the CC_50_ values for HDACis previously tested with primary cell models [33, 34].

**Table 2.** HDAC is showing both less toxicity and higher efficacy

Activator	Structure	(MCF) × (gated percentage)		
		20 µM	2 µM	CC_50_ (µM)*
Chidamide	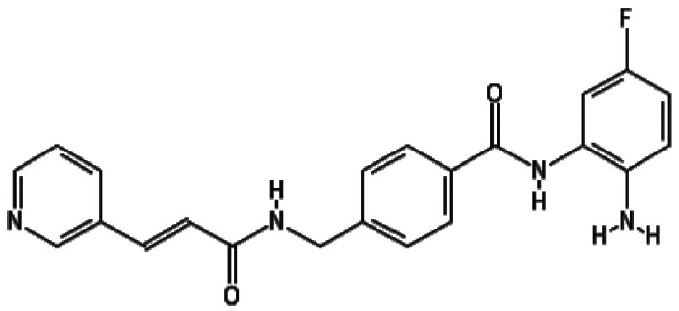	17.08	62.61	92.9 (±35.5)
MS-275	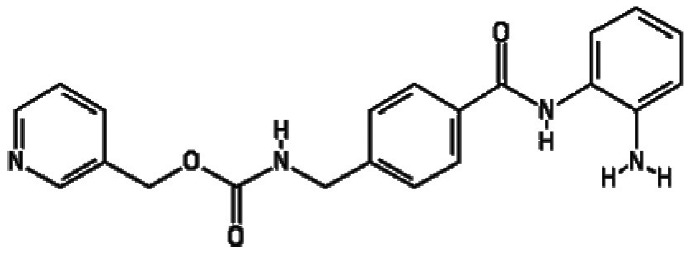	11.21	93.68	91.2 (±20.3)
Pyroxamide	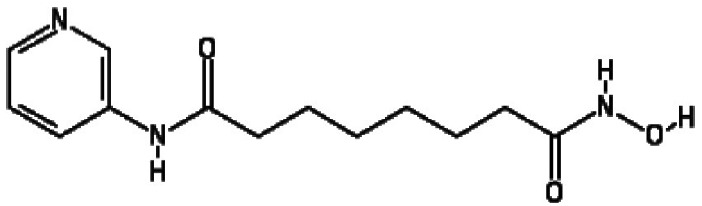	64.90	28.72	81.4 (±32.3)

*CC_50_ was determined by treating resting T cells with the indicated drugs at nine concentrations from 30 nM to 300 µM. Treated cells from four healthy donors were stained with 1.0 µg propidium iodide and analysed with BD Accuri C6. The values obtained were processed with the online software ED50plus v1.0 developed by Dr Mario H. Vergas (Research Unit Instituto Nacional de Enfermedades Respiratorias, MEXICO). Standard deviation is indicated in parentheses next to each CC_50_ value.

### Comparison of the primary cell model to an established cell model of viral latency for LRA activity using the epigenetic modulating compound panel

**Fig. 6.**
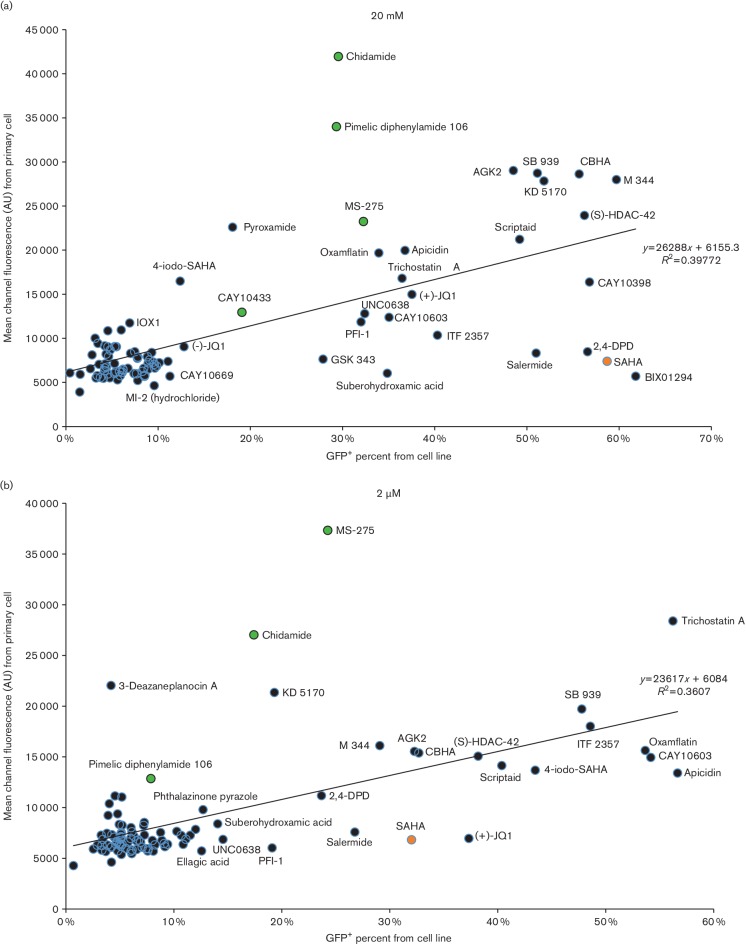
Scatter plot of EGFP MCF from infected primary resting CD4^+^ T cells plotted against GFP^+^ cell percentage of the 24STNLEG cell line treated with drugs in the Epigenetics Screening Library. The data were obtained using cells isolated from three donors compared to a representative experiment from a single culture of the cell line. Graphs are from cells treated with 20 µM for each molecule (a) and 2 µM for each molecule (b). MCF data from resting cells and percentage data from the cell line 24STNLEG are indicated on the *y*-axis and *x*-axis, respectively. Green and orange circles represent HDACis with benzamide groups and SAHA, respectively. In (a), data from chaetocin, UNC1999 and Tenovin-6 are not included because of cell death. AU, arbitrary unit.

A comparison was carried out between the primary cell-based study above and a previously described established cell latency model. For this comparison, the Epigenetics Screening Library was also examined for LRA activity employing the 24STNLEG latency cell model, which is derived from SupT1 cells, an established CD4^+^ T cell line [35]. 24STNLEG cells harbour a latent HIV-1-derived vector virus genome containing the *gfp* gene, so latency antagonist activity was measured by monitoring the change in the percentage of GFP-positive cells. Again, many HDACis distributed in the most active LRA cluster (data not shown). Interestingly, the most potent activators identified were largely different from those obtained when screening with latently infected primary cells. For example, SAHA was quite potent when assayed against 24STNLEG cells, with it being the third and 13th most potent activator at 20 and 2 µM, respectively. However, SAHA barely showed any activity when tested against latently infected primary cells (Fig. 6a, b). Nevertheless, the HDACis with benzamide functional groups, such as MS-275 and chidamide at 2 µM as well as chidamide and pimelic diphenylamide 106 at 20 µM, displayed significantly higher activity with the primary cell model compared to the established cell model (Fig. 6a, b).

## Discussion

In this report, a novel primary cell model of HIV-1 latency was developed. This model was found to display a similar activation profile to samples from patients when treated with a panel of previously identified LRAs, indicating that it could be quite useful in screening compound libraries for LRA drug discovery. This primary cell model was utilized to evaluate a panel of 94 epigenetic modulating compounds. The results suggest that the epigenetic modulating compounds with the greatest potential as LRAs were HDACis containing benzamide groups with pyridine caps, as exemplified by chidamide.

A benzamide functional group is a common functional group among HDACis that chelate Zn^2+^ in the catalytic core of HDACs [36]. Chidamide, MS-275 and pimelic diphenylamide 106 have this functional group, and these HDACis are mostly selective for class I HDACs, including HDAC1, 2, 3 and 8 [36]. Interestingly, it was reported that there is a unique preference of benzamide-containing HDACis for the inhibition of HDAC3 [37], which is highly expressed in resting T cells but not in established T cell lines [34]. The preferential inhibition of an HDAC expressed at high levels in resting T cells could account for the most promising HDACis identified being those containing benzamide groups. It is of note that MS-275 was previously found to display LRA activity in a different primary T cell model of HIV-1 latency [34].

Another intriguing observation is that the HDACis exhibiting activity with the least toxicity had pyridyl residues as their cap group (Table 2), which functions to recognize the surface of target enzymes [38]. Pyridine cap groups are more hydrophilic than phenyl residues found in some HDACis, such as SAHA [39], but their function is not clear. The efficiency of apoptosis induction of HDACis with and without a pyridine cap may help to explain the lower levels of toxicity. Pyroxamide and SAHA are identical except for the cap group with pyroxamide containing a pyridine cap while SAHA contains a phenyl cap. Although both pyroxamide and SAHA can cause apoptosis through HDAC1 and 2 inhibition, it requires an approximately sevenfold higher concentration of pyroxamide than SAHA to induce a similar level of cell death [40]. On the other hand, the *K*_i_ values of pyroxamide against HDAC1–9 are only two- to threefold higher than SAHA [41]. Taken together, these findings suggest the existence of an HDAC1–9-independent apoptotic pathway(s) which HDACis with a pyridine cap, such as pyroxamide, do not activate while HDACis without the pyridine cap, such as SAHA, do activate. To explore more thoroughly the relationship between lower toxicity and pyridine cap-containing HDACis, a detailed analysis of HDAC1–9 independent apoptotic pathways employing the different HDACis would be useful.

To date, clinical trials with HDACis have indicated that HDACis can partially reverse latency but not sufficiently to reduce the size of the latent reservoir [5]. Besides potency, the HDACis utilized to date lack a significant degree of specificity. However, HDACis may prove important for combination therapy aimed at eliminating latent infection. One can imagine a scenario in which HDACis will act synergistically with LRAs that exhibit a high degree of specificity. Because HDACis modify chromatin structure, it follows that they could essentially act as a conditioning molecule that adjusts the chromatin structure thereby increasing the efficacy of more targeted LRAs. Thus, they remain an important class of molecule for further drug discovery to eliminate the latent arm of an HIV-1 infection.

We also found drugs which demonstrated solid activity in both the primary resting cell and the established T cell models (Fig. 4a, b). These molecules included trichostatin A, KD 5170, SB 939, M 344, AGK2 and CBHA, all of which are also HDACis. These compounds mostly contained an aromatic ring and had hydroxamic acid as their functional moiety, except the sirt2 inhibitor AGK2. Most of them displayed only moderate activity on resting cells at the low dose (Fig. 4b) and tended towards higher toxicity, as mentioned above, so these hydroxamic acid-containing HDACis yielded a lower priority score than the benzamide-containing HDACis (Fig. 5a, b). It is noteworthy that a couple of histone methyltransferase inhibitors (HMTis) also displayed promising activity in the primary cell model, namely 3-deazaneplanocin A and UNC1999 (Fig. 5a, b). Previously, 3-deazaneplanocin was found to activate latent HIV-1 in PBMCs [11]. UNC1999 is an inhibitor of the lysine methyltransferases EZH2 and EZH1 and was shown to be orally bioavailable in mice [42], but its effect on HIV-1 activation has not been well studied. More detailed research on these compounds awaits. Additionally, the well-studied HMTi chaetocin did not exhibit significant activity in this evaluation. It is possible that the concentrations of chaetocin were too high in the screen given that the previously reported effective concentration was much lower in the nanomolar range [43].

In summary, a primary cell model of HIV-1 latency, using HIV-1 virions complemented with SIV Vpx to more efficiently transduce resting CD4^+^ T cells, was used to screen an epigenetic compound library for LRA activity. It was found that linear, benzamide HDACis containing a pyridine cap group were particularly effective in primary resting CD4^+^ T cells as latency antagonists. The results suggest that this class of molecule should be considered for further drug development in the quest for a functional cure for HIV-1 infection.

## Methods

### Culture media

All non-adherent cells were cultured in RPMI 1640 GlutaMAX, HEPES medium (Life Technologies) supplemented with 10 % Hyclone FBS (Thermo Scientific Hyclone), MEM non-essential amino acids solution (Life Technologies), and penicillin/streptomycin (100 U ml^−1^/100 µg ml^−1^) solution (Life Technologies). Adherent cells were cultured in DMEM GlutaMAX medium (Life Technologies) supplemented with 10 % FetalClone III Serum (Thermo Scientific Hyclone), MEM non-essential amino acids solution, and 100 U penicillin ml^−1^ plus 100 µg streptomycin ml^−1^ solution.

### Reagents

The following reagents were used: polyethyleneimine (PEI) linear MW 25 kDa (Polysciences), Retro-concentin (System Biosciences), Epigenetics Screening Library (Cayman Chemical), SAHA and raltegravir (Selleckchem), Polybrene, ionomycin, PMA, bryostatin and prostratin (Sigma-Aldrich), JQ1, panobinostat, romidepsin (APExBIO) and Trizol reagent. The High-Capacity cDNA reverse transcription Kit, TaqMan Gene Expression Master Mix (Life Technologies) was also employed. For activation-marker staining, FITC Mouse Anti-Human CD25 (no. 555431; BD Pharmingen), Anti-Human HLA-DR FITC conjugate (no. MHLDR01; Life Technologies) and CD69 Antibody, FITC conjugate (no. MA1–10275; Life Technologies) were used.

### Cell lines

The U251MG cell line H80 was kindly provided by Dr Darell Bigner (Duke University, NC, USA). 24STNLEG cells were produced in this laboratory as described previously [35].

### Isolation of human CD4^+^ T cells

PBMCs were isolated with Histopaque-1077 (Sigma-Aldrich) from leukocyte fractions purchased from the NY Blood Center, and CD4^+^ T cells were purified from them using the Dynabeads Untouched Human CD4^+^ T Cells kit (Life Technologies) with the modification that anti-CD25 antibody was added to the Antibody Mix to remove activated T cells.

### Plasmid constructs

*Gaussia* luciferase and EGFP genes were connected with the T2A self-cleavage sequence [44] and fused to the *nef* gene by overlapping PCR followed by insertion into the *Bam*HI–*Xho*I site of pNL4–3, yielding construct gGn. The SIV_MAC_ Vpx binding motif (DPAVDLL) [19] was introduced into the *Spe*I–*Sbf*I site of gGn Gag p6 by overlapping PCR [21], yielding gGn-p6*. SIV-Vpx expression vector pcDNA-VPXsiv was made by inserting the SIVmac293 Vpx fragment [19], synthesized by Integrated DNA Technologies, into the *Bam*HI–*Xho*I site of pcDNA3.

### Virus production and infection

Virus was produced from 293T cells transfected with both constructs gGn-p6* and pcCNA-VPXsiv using 1 µg PEI per 1 µg DNA. Virus supernatants were filtered with 45 µm filter units and were concentrated 50-fold with Retro-Concentin. Then, 4×10^6^ human CD4^+^ T cells per 500 µl were infected with the gGn-p6* virus containing Vpx, with the concentration equivalent to 0.36 µg p24^gag^ by spinoculation [45] in 24-well plates with 16 µg Polybrene ml^−1^ at 1200 ***g*** for 2 h at 24 °C. Infected cells were incubated for 4 h at 37 °C and further incubated overnight at 37 °C after addition of 2 ml RPMI media. The next day, the medium was changed and co-cultured with H80 cells, unless otherwise stated. All H80 cell cultures were seeded at 0.5×10^6^ ml^−1^2 days before use.

### Titration of virus produced from infected cells

Resting T cells or activated T cells (activated 24 h before virus infection using Dynabeads Human T-Activator CD3/CD28; Life Technologies) were infected with gGn-p6* as described above via spinoculation. More specifically, 4×10^6^ resting T cells or 2×10^6^ activated T cells were infected via spinoculation followed by incubation for 72 h. Cell supernatants were then harvested, serially diluted and used to inoculate fresh HeLaT4 cells (0.5×10^6^ cells) in 24-well plates again via spinoculation. The cells were then washed and incubated for 48 h followed by FACS analysis to determine the number of GFP^+^ HeLaT4 cells. The number of infected GFP^+^ HeLaT4 cells was then normalized to the number of GFP^+^ virus producer cells (resting cells or activated cells).

### Infected cell analysis

Infected CD4^+^ T cells were incubated for 72 h and treated with 10 µM raltegravir and test reagents for 48 h. All the flow cytometric analyses were done with an Accuri C6 flow cytometer (BD Biosciences). Contamination with lifted H80 cells was excluded by gating according to cell size and granularity.

### HIV-1 RNA quantification

Relative HIV-1 mRNA amounts were determined by using a modification of the method described by Bullen *et al.* [46]. RNA from 3–5×10^5^ resting T cells was isolated using Trizol reagent and cDNAs were produced from the RNAs with both random hexamer and oligo-dT using the High-Capacity cDNA reverse transcription Kit (Life Technologies). Quantitative PCR was performed with TaqMan Gene Expression Master Mix using primers CAGATGCTGCATATAAGCAGCTG and TTTTTTTTTTTTTTTTTTTTTTTTGAAGCAC and probe FAM-CCTGTACTGGGTCTCTCTGG-Iowa black. After 40 cycles of amplification, the 2^–Ct^ value from each sample was normalized by GFP^+^ cell number measured by flow cytometry. The values obtained were then normalized to values obtained from the mock-treated cells.

### Library screening procedure

In total, 0.5–1.0×10^5^ resting cells transduced with gGn-p6* in 100 µl media were transferred to 96-well plates. MCF was obtained via flow cytometry using a BD Accuri C6 instrument (BD Biosciences) 48 h after the addition of test compound.

### CC_50_ determination

CC_50_ was determined for each test drug by treating resting CD4^+^ T cells with 30 nM, 100 nM, 300 nM, 1 µM, 3 µM, 10 µM, 30 µM, 100 µM and 300 µM of the tested drug. Treated cells from four healthy donors were stained with 1.0 µg propidium iodide and analysed via flow cytometry. The values obtained were processed with the software ED50plus v1.0 (Mario H. Vergas, 2000).
